# Pro‐Inflammatory Dietary Patterns Are Associated With Atopic but Not Non‐Atopic Dermatitis in Asian Adults: Evidence From a Cross‐Sectional Study

**DOI:** 10.1111/cea.70232

**Published:** 2026-02-08

**Authors:** Jun Jie Lim, Jia Yi Karen Wong, Zongxun Huang, Kavita Reginald, Yee‐How Say, Mei Hui Liu, Fook Tim Chew

**Affiliations:** ^1^ Department of Biological Sciences, Faculty of Science National University of Singapore Singapore Singapore; ^2^ Department of Biomedical Sciences, Sir Jeffrey Cheah Sunway Medical School, Faculty of Medical and Life Sciences Sunway University Petaling Jaya Malaysia; ^3^ Department of Biomedical Science, Faculty of Science Universiti Tunku Abdul Rahman (UTAR) Kampar Perak Malaysia; ^4^ Department of Food Science & Technology, Faculty of Science National University of Singapore Singapore Singapore

**Keywords:** atopic dermatitis, dietary indices, Dietary Inflammatory Index, epidemiology, inflammation, prevention

## Abstract

- Pro‐inflammatory diets were associated with higher odds of atopic dermatitis (AD), but not non‐atopic dermatitis, suggesting specificity to atopic mechanisms.
- Directionally consistent associations observed using both a nutrient‐based (Dietary Inflammatory Index) and a food‐based (Mediterranean‐like) dietary score support the potential robustness of diet‐AD relationships.

To the Editor,

Diet is a potentially modifiable risk factor for atopic dermatitis (AD), yet evidence on healthy dietary patterns in AD is limited, as most studies have understandably focused on individual foods or nutrients rather than habitual diet. The Dietary Inflammatory Index (DII) quantifies the pro‐ versus anti‐inflammatory potential of the overall diet and has been associated with several allergic and inflammatory conditions [1, 2, 3]. However, data on adult AD, particularly in Asian populations, remain scarce. Furthermore, it is unclear whether diet–dermatitis associations are specific to atopic mechanisms or reflect more general skin inflammation, and few studies have assessed whether findings are consistent across alternative dietary pattern indices. To address these gaps, we primarily examined the association between DII and dermatitis phenotypes in a large, well‐characterised Asian adult cohort, comparing atopic and non‐AD phenotypes. Secondarily, we assessed the robustness and interpretability of findings using a complementary, food‐based modified Mediterranean Diet Score (MDS) [4].

We analysed 13,011 young Chinese adults from the Singapore/Malaysia Cross‐sectional Genetics Epidemiology Study (SMCGES). Habitual dietary intake over the preceding 12 months was assessed using a validated, investigator‐administered semi‐quantitative food frequency questionnaire (FFQ), covering 16 major food groups [5]. Nutrient intakes were estimated from these food groups using the United States Department of Agriculture FoodData Central database, and DII scores were calculated following the standardised method. Positive DII values indicate pro‐inflammatory diets, negative values reflect anti‐inflammatory diets, and values at zero represent a neutral inflammatory potential [1]. While the original DII includes up to 45 dietary parameters [1], prior validation shows that calculating the DII from 26 nutrients maintains robustness and predictive performance comparable to that of the full DII [2, 6]. Participants were categorised into quartiles (Q1–Q4) based on the distribution of DII scores, with Q1 (−4.58 to < −0.12) representing the most anti‐inflammatory diets, Q2 (≥ −0.12 to < 0.78) moderately anti‐inflammatory, Q3 (≥ 0.78 to < 1.60) approaching pro‐inflammatory and Q4 (≥ 1.60 to 4.50) the most pro‐inflammatory. An adapted MDS was additionally derived to reflect relative adherence to a Mediterranean‐like dietary pattern within this cohort.

In this study, AD was defined using a combined clinical‐atopic approach requiring both recurrent itchy flexural rash (assessed per Hanifin and Rajka and UK Working Party criteria, and cross‐validated by dermatologists) and objective atopy (IgE‐mediated house dust mite sensitisation [HDM]) [7]. Participants without HDM sensitisation and no history of dermatitis were classified as non‐AD controls, and non‐AD (itchy flexural rash without atopy) was analysed separately. The cohort comprised 2,321 ad cases (17.8%), 980 non‐AD cases (7.5%), and 3,571 non‐AD controls (27.4%). Associations between dietary indices and dermatitis outcomes were evaluated using multivariable logistic regression models adjusted for sex, age (years), body mass index (BMI) (Asian classification), physical activity, household income, parental AD and total energy intake (kcal/week) (Figure 1).

**FIGURE 1:**
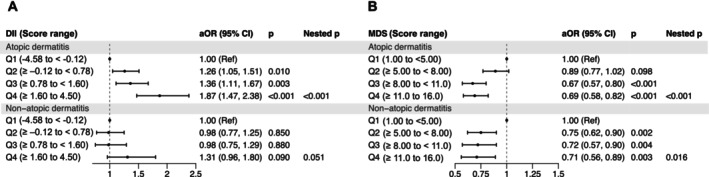
Forest plots of the association between the dietary indices and dermatitis phenotypes in the SMCGES cohort. (A) Modified Dietary Inflammatory Index (DII) was analysed as a categorical variable in two comparisons: Participants with atopic dermatitis (AD) (*n* = 2321) versus non‐AD controls (*n* = 3571), and non‐AD (*n* = 980) versus non‐AD controls (*n* = 3571). (B) Association between the Modified Mediterranean Diet Score (MDS) and dermatitis phenotypes using the same analytic groups. The dotted line at adjusted odds ratio (aOR) = 1.00 denotes the reference. aORs with 95% confidence intervals (CIs) and *p* values are presented. Multivariable models were adjusted for sex, body mass index (Asian classification), physical activity, household income, parental history of AD and total energy intake (kcal/week).

Baseline characteristics were broadly similar across groups; however, participants with dermatitis showed slightly higher energy intake and BMI, and a greater prevalence of overweight and parental AD history than non‐AD controls, while those with AD showed modestly higher household income and outdoor physical activity. DII scores ranged from −4.58 to 4.50 (mean 0.63 ± 1.45), indicating generally mildly pro‐inflammatory dietary patterns (repository). Higher DII was associated with increased odds of AD in a dose‐dependent manner. Compared with Q1, participants in Q2 (adjusted odds ratio [aOR]: 1.26, 95% confidence interval [CI]: 1.05–1.51, *p* = 0.014), Q3 (aOR: 1.36, 95% CI: 1.11–1.67, *p* = 0.004) and Q4 (aOR = 1.87, 95% CI: 1.47–2.38, *p* < 0.001), all had significantly higher odds of AD. No significant associations were observed for non‐AD with DII (Figure 1A), suggesting specificity of the DII–AD relationship.

In contrast, higher adherence to a Mediterranean‐like diet was associated with lower odds of AD, with similar significant effect emerging from Q3 (≥ 8.00 to < 11.0) (aOR: 0.67, 95% CI: 0.57–0.80, *p* < 0.001) and Q4 (≥ 11.0 to 16.0) (aOR: 0.69, 95% CI: 0.58–0.82, *p* < 0.001), but not at Q2 (≥ 5.00 to < 8.00), suggesting a threshold effect. For non‐AD, MDS showed a modest but consistent inverse dose–response association across quartiles (Figure 1B). Together, these findings indicate that a pro‐inflammatory nutrient profile, captured by the DII, is more strongly and specifically associated with AD, whereas an overall food‐based dietary quality is associated with broader skin health. The concordant findings across two conceptually distinct dietary indices support the robustness of the observed diet–AD association.

A core limitation of the DII is that it was developed using systemic inflammatory markers (e.g., IL‐6, CRP, TNF‐α) rather than skin‐specific endpoints, potentially overlooking nutrients that directly influence barrier function, lipid metabolism or the cutaneous microbiome. Nevertheless, its dermatological relevance is supported by associations with psoriasis and acne, suggesting potential links between systemic inflammation and cutaneous immunity [8, 9]. As the DII was largely derived from Western dietary data, culturally specific components common in Asian diets, such as sodium‐rich or fermented foods, may be under‐represented. Additional indices, including Dietary Approaches to Stop Hypertension (DASH) or Healthy Eating Index (HEI), could further strengthen the inference but require quantitative portion‐size data not captured by the current frequency‐based FFQ. Strengths of our study include detailed nutrient profiling, prior FFQ validation against food diaries, and a clinically well‐characterised cohort. Future work should develop culturally relevant dietary metrics, integrate skin‐targeted biomarkers, and apply longitudinal or interventional designs to clarify causal relationships with AD.

## Author Contributions

F.T.C. conceived and supervised the research study, and critically reviewed the manuscript. J.J.L. conducted the literature review, drafted the manuscript and, together with J.Y.K.W., planned and interpreted the statistical analyses. J.J.L., J.Y.K.W. and Z.H. were responsible for figure preparation and data visualisation. J.J.L., J.Y.K.W., Z.H., K.R. and Y.‐H.S. collected the data. M.H.L. provided expert input on the manuscript. All authors read and approved the final manuscript for submission.

## Funding

Fook Tim Chew (F.T.C.) received grants from the National University of Singapore (N‐154‐000‐038‐001 (E‐154‐00‐0017‐01); C141‐000‐077‐001 (E‐141‐00‐0096‐01)), Singapore Ministry of Education Academic Research Fund (R‐154‐000‐191‐112; R‐154‐000‐404‐112; R‐154‐000‐553‐112; R‐154‐000‐565‐112; R‐154‐000‐630‐112; R‐154‐000‐A08‐592; R‐154‐000‐A27‐597; R‐154‐000‐A91‐592; R‐154‐000‐A95‐592; R‐154‐000‐B99‐114), Biomedical Research Council (BMRC) (Singapore) (BMRC/01/1/21/18/077; BMRC/04/1/21/19/315; BMRC/APG2013/108), Singapore Immunology Network (SIgN‐06‐006; SIgN‐08‐020), National Medical Research Council (NMRC) (Singapore) (NMRC/1150/2008; OFIRG20nov‐0033; MOH‐001636 (OFLCG23may‐0038, A‐8002641‐00‐00)), National Research Foundation (NRF) (Singapore) (NRF‐MP‐2020‐0004), Singapore Food Agency (SFA) (SFS_RND_SUFP_001_04; W22W3D0006; NRF‐SFSRND2SIH‐0001), Singapore's Economic Development Board (EDB) (A‐8002576‐00‐00), and the Agency for Science Technology and Research (A*STAR) (Singapore) (H17/01/a0/008; and APG2013/108). This research is supported by the National Research Foundation Singapore under its Open Fund‐Large Collaborative Grant (MOH‐001636) (A‐8002641‐00‐00) and administered by the Singapore Ministry of Health's National Medical Research Council. Kavita Reginald (K.R.) has received funding from the T20 Research Collaboration Grant Scheme from Sunway University with Grant No.: STR‐RMF‐T20‐005‐2019. The funding agencies had no role in the study design, data collection and analysis, decision to publish or preparation of the manuscript.

## Ethics Statement

The study was conducted in accordance with the ethical guidelines outlined in the Declaration of Helsinki, Good Clinical Practice, and applicable local regulations. Ethical approval was received from the Institutional Review Board of the National University of Singapore (approval IRB codes NUS‐07‐023, NUS‐09‐256, NUS‐10‐445, NUS‐13‐075, NUS‐14‐150 and NUS‐18‐036), the Scientific and Ethical Review Committee of Universiti Tunku Abdul Rahman (reference code U/SERC/03/2016) and the Research Ethics Committee of Sunway University (reference code SUREC 2019/029). Written informed consent was explicitly obtained from all participants prior to their inclusion in the study through an approved Participant Information Sheet and Consent Form. For participants under the legal age of 21 in Singapore and 18 in Malaysia, parental or guardian consent was also acquired.

## Conflicts of Interest

F.T.C. reports grants from the National University of Singapore, Singapore Ministry of Education Academic Research Fund, Singapore Immunology Network, National Medical Research Council (NMRC) (Singapore), Biomedical Research Council (BMRC) (Singapore), National Research Foundation (NRF) (Singapore), Singapore Food Agency (SFA), Singapore's Economic Development Board (EDB) and the Agency for Science Technology and Research (A*STAR) (Singapore) during the conduct of the study; and consulting fees from Sime Darby Technology Centre, First Resources Ltd., Genting Plantation, Olam International, Musim Mas and Syngenta Crop Protection, outside the submitted work. The other authors declare no other competing interests.

## Data Availability

The data that support the findings of this study are openly available in Dietary Inflammatory Index Analysis at https://osf.io/gdcqj/overview?view_only=add6a362cf244764a0553eee8a893bd9. Further data are available upon reasonable request from the corresponding author (F.T.C.).

## References

[1] N. Shivappa , S. E. Steck , T. G. Hurley , J. R. Hussey , and J. R. Hébert , “Designing and Developing a Literature‐Derived, Population‐Based Dietary Inflammatory Index,” Public Health Nutrition 17, no. 8 (2014): 1689–1696, 10.1017/S1368980013002115. 23941862 PMC3925198

[2] H. Shen , J. Liao , L. Zhang , et al., “Association Between the Dietary Inflammatory Index and Allergic Rhinitis Results From the National Health and Nutrition Examination Survey (2005‐2006),” Journal of Health, Population, and Nutrition 44, no. 1 (2025): 179, 10.1186/s41043-025-00932-0. 40442763 PMC12124075

[3] E. Visser , K. de Jong , T. van Zutphen , H. A. M. Kerstjens , and A. Ten Brinke , “Dietary Inflammatory Index and Clinical Outcome Measures in Adults With Moderate‐To‐Severe Asthma,” Journal of Allergy and Clinical Immunology. In Practice 11, no. 12 (2023): 3680–3689.e7, 10.1016/j.jaip.2023.08.032. 37652347

[4] M. A. Martínez‐González , M. García‐López , M. Bes‐Rastrollo , et al., “Mediterranean Diet and the Incidence of Cardiovascular Disease: A Spanish Cohort,” Nutrition, Metabolism, and Cardiovascular Diseases: NMCD 21, no. 4 (2011): 237–244, 10.1016/j.numecd.2009.10.005. 20096543

[5] P. Ellwood , M. I. Asher , L. García‐Marcos , et al., “Do Fast Foods Cause Asthma, Rhinoconjunctivitis and Eczema? Global Findings From the International Study of Asthma and Allergies in Childhood (ISAAC) Phase Three,” Thorax 68, no. 4 (2013): 351–360, 10.1136/thoraxjnl-2012-202285. 23319429

[6] N. Shivappa , J. R. Hebert , A. Marcos , et al., “Association Between Dietary Inflammatory Index and Inflammatory Markers in the HELENA Study,” Molecular Nutrition & Food Research 61, no. 6 (2017): 1600707, 10.1002/mnfr.201600707. PMC5517083 27981781

[7] J. J. Lim , K. Reginald , Y. H. Say , M. H. Liu , and F. T. Chew , “Associations Between Self‐Reported Dietary Intake and Atopic Dermatitis Risk in Young Adults From Singapore and Malaysia,” Clinical and Experimental Allergy: Journal of the British Society for Allergy and Clinical Immunology 55, no. 6 (2025): 493–495, 10.1111/cea.14629. 39838549 PMC12127055

[8] A. Kashani , J. Moludi , H. Lateef Fateh , A. Tandorost , H. Jafari‐Vayghan , and P. Dey , “Dietary Inflammatory Index in Relation to Psoriasis Risk, Cardiovascular Risk Factors, and Clinical Outcomes: A Case‐Control Study in Psoriasis Patients,” Applied Physiology, Nutrition, and Metabolism 46, no. 12 (2021): 1517–1524, 10.1139/apnm-2021-0217. 34348057

[9] J. Burris , J. Shikany , W. Rietkerk , and K. Woolf , “Dietary Inflammatory Index Is Associated With Moderate and Severe Acne in Adults,” Journal of the Academy of Nutrition and Dietetics 119, no. 10 (2019): A110.

